# A Comparative Study of Coupled Preferential Crystallizers for the Efficient Resolution of Conglomerate-Forming Enantiomers

**DOI:** 10.3390/pharmaceutics9040055

**Published:** 2017-12-05

**Authors:** Aniruddha Majumder, Zoltan K. Nagy

**Affiliations:** 1 School of Engineering, University of Aberdeen, Aberdeen AB24 3UE, UK; 2 Department of Chemical Engineering, Loughborough University, Loughborough LE11 3TU, UK; zknagy@purdue.edu; 3 Davidson School of Chemical Engineering, Purdue University, West Lafayette, IN 47907-2100, USA

**Keywords:** chiral resolution, coupled crystallizer, crystallization modelling, enantioseparation, MSMPR

## Abstract

The separation of enantiomers is of great importance due to their possible differences in therapeutic properties. Preferential crystallization in various configurations of coupled batch crystallizers is used as an attractive means to separate the conglomerate-forming enantiomers from racemic mixtures. However, the productivity of such batch processes can be limited by the nucleation of the counter enantiomer and consumption of the supersaturation. In this work, a recently proposed process configuration, which uses coupled mixed suspension mixed product removal (MSMPR) with liquid phase exchange, is investigated by simulation studies. A detailed study on the effect of process parameters (e.g., feed flow rate, seed mass, and liquid phase exchange) on the productivity and yield of the coupled MSMPR has been presented. Moreover, a comparison of various coupled crystallizer configurations is carried out. It is shown through simulation studies that the productivity of the enantiomeric separation can be significantly improved compared to the previously proposed batch modes when the continuous configuration is used. The effect of nucleation kinetic parameters on the performances of various crystallizer configurations is studied as well. A set of coupled population balance equations (PBEs) was used to describe the evolution of the crystal phase of the both enantiomers in each vessel. These equations were solved numerically using the quadrature method of moments. The insights obtained in this study will be useful in the process design of coupled crystallizer systems.

## 1. Introduction

Enantiomers are the result of the chirality or handedness of molecules which are the non-superimposable mirror images of each other. Enantiomers are widely found in organic compounds and their separation is a major concern in the modern pharmaceutical, food, and agricultural industries. Separation of the enantiomorphs is particularly important in pharmaceutical industry, since very often one of the enantiomers exhibits the intended therapeutic activity while the other is inert. Moreover, in some cases the counter enantiomer can cause toxicity or adverse effects. For example, *S*(−)-fluoxetine shows remarkable therapeutic effects in preventing migraines, while the racemic (equimolar mixture of both enantiomers) drug (the antidepressant Prozac) has no effect [1]. Thalidomide consisting of *S*(−) and *R*(+) enantiomers can interconvert under physiological conditions. *R*(+)-thalidomide seems to act as a sedative while *S*(−)-thalidomide and its derivatives are reported to be teratogenic (causing malformations of an embryo or fetus) [2]. Thus, it is demanded by the regulating authorities that the chiral drugs are administered in an optically pure form [3]. This stringent regulatory policy has intensified efforts in industrial and academic research to develop processes that are able to produce pure enantiomers.

There are two major strategies to obtain a single pure enantiomer: (1) the ‘chiral approach’ is based on developing the asymmetric synthesis of the selected enantiomer; and (2) the ‘racemic approach’ is based on the separation of the mixture of two enantiomers [4]. Although ideal, the asymmetric synthesis route can be time-consuming to develop. Moreover, the number of highly selective reactions that supply pure enantiomers on an industrially relevant scale is still limited [4]. Conversely, the latter is the most widely used approach to produce preferred enantiomers, since the enantiomers are typically readily available in racemic composition. Various means available for the resolution of enantiomers include classical chiral separation techniques such as preferential crystallization [5,6,7,8,9,10,11,12,13], Viedma ripening [14,15,16], chromatography [1,17,18,19], and membrane separation [20,21]. Some hybrid techniques coupling chromatography and crystallization are also proposed [22,23,24]. A more detailed review of the various techniques can be found in Lorenz and Seidel-Morgenstern [4].

Among various means, preferential crystallization is an attractive and efficient way to separate the racemic mixtures since no auxiliaries and reagents other than solvent are needed [10]. However, preferential crystallization can only be used for conglomerate-forming systems (which make up 10% of the available enantiomers [5]). In such systems the stable crystalline phases consist of either the pure *S* enantiomer or pure *R* enantiomer. This is in contrast to the racemic compound-forming system where there is an additional racemic phase consisting of equal amounts of *R* and *S* enantiomers. Crystallization can be carried out in standard equipment readily available in pharmaceutical and fine chemical industries. Manufacturing methods of the enantiomorphic drug are still dominated by crystallization [4]. In preferential crystallization, the process is operated in the metastable zone, i.e., a region in the phase diagram where the solution is thermodynamically unstable but kinetically inhibited based on different initial surface areas [25]. Addition of the homochiral seeds at this stage favours the crystallization of a particular enantiomer which can be harvested as solid phase thus providing enantioseparation. In particular, it has been shown that batch coupled preferential crystallizer (CPC) with liquid exchange can be used as an attractive means of enantioseparation [9,10,25]. However, the productivity of the regular CPC is limited by the depletion of the supersaturation. Moreover, it has been shown that a variant of the CPC configuration, coupled preferential crystallization-dissolution (CPC-D), can increase the productivity [11,26]. This alternative configuration is based on coupling preferential crystallization in one vessel, with selective dissolution of the solid racemate in the other. However, in this process configuration, one needs to collect the pure crystals within a window of time determined by the appearance of the counter enantiomer in the crystallization vessel and complete dissolution of one enantiomer in the dissolution vessel [11,26].

Recently, a continuous version of the CPC configuration was proposed [27,28,29]. This configuration utilizes two mixed suspension mixed product removal (MSMPR) crystallizers which are coupled through solid free liquid phase exchange, henceforth referred as CPC-MSMPR. Theoretical and experimental investigations demonstrate the efficiency and increased productivity of the CPC-MSMPR configuration [27,28,30]. However, a thorough comparison of the various coupled crystallizer configurations for the separation of enantiomorphs has not been performed. The objective of this study is to compare the performance of CPC, CPC-D, and CPC-MSMPR in terms of productivity and yield. Furthermore, the effect of various process parameters (e.g., feed flow rate, seed mass, and liquid phase exchange rate) on the productivity and yield of the CPC-MSMPR is studied. The details of the coupled crystallizer configurations are discussed next.

## 2. Coupled Preferential Crystallizer Configurations

### 2.1. CPC-MSMPR Configuration

**Figure 1:**
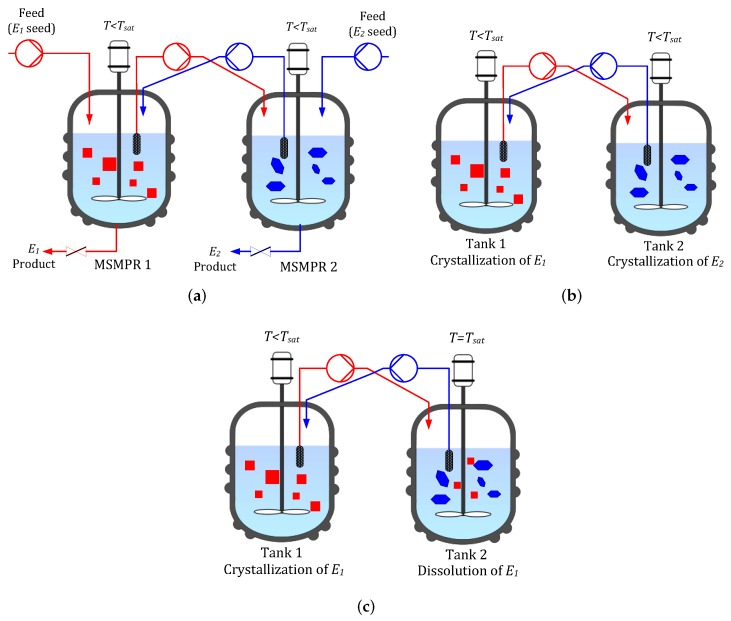
(**a**) Coupled preferential crystallization in MSMPR crystallizers (CPC-MSMPR), where each vessel initially containing supersaturated racemic solution is continuously fed with a slurry containing the seed of the specific enatiomorph. Exchange of liquid phase makes use of the depletion of the counter enantiomer in the other vessel; (**b**) The coupled preferential crystallizer (CPC) configuration, which is similar to CPC-MSMPR except that there is no continuous feed and product removal; (**c**) The coupled preferential crystallization-dissolution (CPC-D) configuration, which is similar to CPC except that Tank 2 (seeded with racemic solid) is maintained at saturation temperature so that dissolution of counter enantiomorph $E_{1}$ takes place.

As shown in Figure 1a, the process configuration involves two mixed suspension mixed product removal (MSMPR) crystallizers maintained at a temperature lower than the saturation temperature and coupled through liquid phase exchange. The MSMPR crystallizers are mixed well enough to ensure uniform conditions. The process starts with a supersaturated racemic solution in both the vessels. Slurry containing racemic solution and seeds of the opposite homochiral enantiomers is continuously fed into two different vessels. The continuous supply of seeds can be maintained by ultrasonic comminution of crystals, withdrawn from the bottom of the MSMPR crystallizers [12]. This slurry addition allows growth and nucleation of the opposite enantiomers in the different MSMPR crystallizers while almost completely suppressing the nucleation of the counter enantiomers. The equal volume of slurry will be continuously taken out of each crystallizer as a product stream in order to maintain the same slurry volume within the crystallizers. Due to selective crystallization in each vessel, e.g., $E_{1}$ in Tank 1 and $E_{2}$ in Tank 2, the concentration of the preferred enantiomer in the liquid phase decreases. The liquid phase exchange between the vessels makes up this depletion of the preferred enantiomer to some extent while reducing the concentration of the counter enantiomer, and thus helps to prevent the primary nucleation of counter enantiomer.

### 2.2. CPC Configuration

This configuration, shown in Figure 1b, is the batch version of the CPC-MSMPR, i.e., there are no continuous feed and product removals. The process starts when homochiral seeds are introduced in the vessels containing racemic solution at a temperature lower than the saturation temperature (i.e., in the metastable zone). As a result, preferential crystallization of seeded enantiomers takes place in each tank. The solid free liquid phases are exchanged between the two vessels. The process can be run until the supersaturation is depleted or the counter enantiomorph appears in any vessel due to nucleation. The product crystals are obtained at the end of the batch.

### 2.3. CPC-D Configuration

This configuration is run in batch mode. However, the two tanks are maintained at two different temperatures as shown in Figure 1c. At first, both the tanks are fed with racemic liquid solution. The temperature of Tank 1 is lower than the saturation temperature but within the metastable zone. Tank 2 is maintained at the saturation temperature and a racemic solid mixture is added to Tank 2. The process begins when Tank 1 is seeded with enantiopure seeds, $E_{1}$. Since the liquid phase is supersaturated in Tank 1, preferential crystallization of the seeded enantiomorph $E_{1}$ takes place and solid mass increases. Due to the exchange of solid free liquid phase, the solution in Tank 2 becomes under-saturated with respect to $E_{1}$. This leads to the selective dissolution of $E_{1}$ in Tank 2. As a result, concentration of $E_{1}$ in Tank 2 increases until saturation concentration is reached. The liquid phase exchange also ensures that depletion of $E_{1}$ in Tank 1 is partially compensated. The counter enantiomer $E_{2}$ remains in solid form in Tank 2 as the concentration of $E_{2}$ is not affected by the crystallization of $E_{1}$. The process can be run until primary nucleation for $E_{2}$ is detected in Tank 1 or dissolution of $E_{1}$ is complete in Tank 2.

In this study, the preferential crystallization of amino acid threonine dissolved in water is used as a model system. The process is modeled using widely used population balance equation (PBE) [28,30] coupled with the mass balance equation. The required growth and nucleation kinetic parameters are experimentally estimated and validated [10,11], and are presented in tabular form in the Appendix.

## 3. Model of the Coupled Crystallization Process

The model presented here is based on Eicke et al. [11] and has been verified experimentally. In a crystallizer, crystals are dispersed in a continuous phase or solution phase. The population balance equation (PBE) is used to describe the evolution of the crystal size distribution (CSD) in MSMPR as follows

(1) $$\begin{array}{c} \frac{\partial f_{k}^{\left(j\right)}}{\partial t}+\frac{\partial }{\partial L}\left(R_{k}^{\left(j\right)}f_{k}^{\left(j\right)}\right)=\frac{q}{V}\left(f_{k,feed}^{\left(j\right)}-f_{k}^{\left(j\right)}\right);\;j\in \left\{T1,T2\right\},\;k\in \left\{E_{1},E_{2}\right\}, \end{array}$$

where $f_{k}^{\left(j\right)}$ is the number density or CSD of enantiomer *k* in Tank *j* where T1 and T2 stand for Tank 1 and Tank 2, respectively, *L* is the characteristic size of the crystals, $R_{k}^{\left(j\right)}$ is either the size-dependent growth rate, $G_{k}^{\left(j\right)}$, or dissolution rate, $D_{k}^{\left(j\right)}$, depending on the degree of supersaturation, $S_{k}^{\left(j\right)}$

(2) $$R_{k}^{\left(j\right)}=\left\{\begin{array}{cc} G_{k}^{\left(j\right)} & \mathrm{for}\;S_{k}^{\left(j\right)}>1,\\ 0 & \mathrm{for}\;S_{k}^{\left(j\right)}=0,\\ D_{k}^{\left(j\right)} & \mathrm{for}\;S_{k}^{\left(j\right)}<1, \end{array}\right.\;j\in \left\{T1,T2\right\},\;k\in \left\{E_{1},E_{2}\right\},$$

*q* is the volumetric flow rate for the feed (q=0 for batch configuration) and product stream, and *V* is the volume of the crystallizer. The necessary initial and boundary conditions required are

(3) $$\begin{array}{c} f_{k}^{\left(j\right)}{\left(L,t\right)|}_{t=0}=f_{k,seed}^{\left(j\right)}\left(L,t\right), \end{array}$$

(4) $$\begin{array}{c} f_{k}^{\left(j\right)}{\left(L,t\right)|}_{L=L_{0}}=\left\{\begin{array}{cc} \frac{B_{k}^{\left(j\right)}}{G_{k}^{\left(j\right)}} & \mathrm{for}\;S_{k}^{\left(j\right)}\geq 1,\\ 0 & \mathrm{for}\;S_{k}^{\left(j\right)}<1, \end{array}\right.\;j\in \left\{T1,T2\right\},\;k\in \left\{E_{1},E_{2}\right\}, \end{array}$$

where $B_{k}^{\left(j\right)}$ is the rate of nucleation which is assumed to be taking place at size $L_{0}$. It is further assumed that the particles vanish at size $L_{0}$ during dissolution. In this technique, both the MSMPR crystallizers are fed with one pure enantiomer of an opposite kind

(5) $$\begin{array}{c} f_{k,feed}^{\left(T1\right)}=\left\{\begin{array}{cc} f_{E_{1,seed}}^{\left(T1\right)} & \mathrm{for}\;k=E_{1},\\ 0 & \mathrm{for}\;k=E_{2}, \end{array}\right. \end{array}$$

(6) $$\begin{array}{c} f_{k,feed}^{\left(T2\right)}=\left\{\begin{array}{cc} 0 & \mathrm{for}\;k=E_{1},\\ f_{E_{2,seed}}^{\left(T2\right)} & \mathrm{for}\;k=E_{2}, \end{array}\right. \end{array}$$

The measured CSDs of threonine-controlled samples (sample size >500 particles) can be approximated by a log-normal distribution with sufficient accuracy in terms of the characteristic length

(7) $$\begin{array}{c} f_{k,seed}^{\left(j\right)}=A_{k,seed}^{\left(j\right)}\frac{1}{\sqrt{2\pi }\sigma_{\ln,k}^{\left(j\right)}L}\exp\left(-\frac{1}{2}{\left[\ln\left(\frac{L}{L_{mean,k}^{\left(j\right)}}\right)\frac{1}{\sigma_{\ln,k}^{\left(j\right)}}\right]}^{2}\right). \end{array}$$

The parameter $A_{k,seed}^{\left(j\right)}$ is the ratio of the mass of seeds and the mass of the control sample given by

(8) $$\begin{array}{c} A_{k,seed}^{\left(j\right)}=\frac{m_{k,seed}^{\left(j\right)}}{k_{v}\rho_{s}\int_{0}^{\infty }L^{3}f_{k,cs}^{\left(j\right)}dL}=\frac{m_{k,seed}^{\left(j\right)}}{k_{v}\rho_{s}\mu_{3,k,cs}^{\left(j\right)}}, \end{array}$$

where $f_{k,cs}^{\left(j\right)}$ and $\mu_{3,k,cs}^{\left(j\right)}$ are the CSD and third moment of the control sample, respectively.

In order to track the liquid phase concentration the mass balance has to be considered as follows

(9) $$\begin{array}{c} \frac{dm_{k,L}^{\left(j\right)}}{dt}=F_{ex}\rho_{L}^{\left(j*\right)}w_{k,L}^{\left(j*\right)}-F_{ex}\rho_{L}^{\left(j\right)}w_{k,L}^{\left(j\right)}-3k_{v}\rho_{s}\int_{0}^{\infty }L^{2}R_{k}^{\left(j\right)}f_{k}^{\left(j\right)}dL\\ +q\rho_{L,feed}^{\left(j\right)}w_{k,L,feed}^{\left(j\right)}-q\rho_{L}^{\left(j\right)}w_{k,L}^{\left(j\right)},\\ j*\neq j\;\mathrm{with}\;j,j*\in \{T1,T2\} \end{array}$$

where $m_{k,L}^{\left(j\right)}$ is the mass of enantiomorph *k* at the liquid phase in Tank *j*, $F_{ex}$ is the volumetric liquid phase exchange rate, $\rho_{L}$ is the current density of the liquid phase, and $w_{k,L}^{\left(j\right)}$ is the mass fraction of each component defined as

(10) $$\begin{array}{c} w_{k,L}^{\left(j\right)}=\frac{m_{k,L}^{\left(j\right)}}{\sum_{k}m_{k,L}^{\left(j\right)}+m_{H_{2}O}}. \end{array}$$

The mass of water $m_{H_{2}O}$ is assumed to be constant in both the tanks. The density of the liquid phase can be calculated by an empirical formula taking into account the composition as follows

(11) $$\begin{array}{c} \rho_{L}^{\left(j\right)}\left(t\right)=\rho_{H_{2}O}^{\left(j\right)}\left(T^{\left(j\right)}\right)+K_{3}\sum_{k}w_{L}^{\left(j\right)}\left(t\right), \end{array}$$

(12) $$\begin{array}{c} \rho_{H_{2}O}^{\left(j\right)}\left(T^{\left(j\right)}\right)=\frac{1}{K_{1}+K_{2}{\left(T^{\left(j\right)}\right)}^{2}}, \end{array}$$

where $\rho_{H_{2}O}^{\left(j\right)}$ denotes the density of water at a given temperature and $K_{1}$, $K_{2}$, and $K_{3}$ are constants. The supersaturation driving force is expressed as a ratio of the present mass fraction and the equilibrium mass fraction as

(13) $$\begin{array}{c} S_{k}^{\left(j\right)}\left(t\right)=\frac{w_{k,L}^{\left(j\right)}\left(t\right)}{w_{k,L,eq}^{\left(j\right)}\left(T^{\left(j\right)}\right)}. \end{array}$$

The solubility of the DL-threonine in water for a relatively wide temperature and composition range is given by the following expression

(14) $$\begin{array}{c} w_{k,L,eq}^{\left(j\right)}\left(T^{\left(j\right)}\right)=a_{2sol}{\left[w_{k*,L}^{\left(j\right)}\left(t\right)\right]}^{2}+a_{1sol}w_{k*,L}^{\left(j\right)}\left(t\right)+m_{sol}T+b_{sol}, \end{array}$$

(15) $$\begin{array}{c} k*\neq k\;\mathrm{with}\;k,k*\in \{E_{1},E_{2}\}. \end{array}$$

The empirical size-dependent growth rate expression is found to be as follows

(16) $$\begin{array}{c} G_{k}^{\left(j\right)}\left(t,L\right)=k_{g}^{\left(j\right)}\left(T^{\left(j\right)}\right){\left[S_{k}^{\left(j\right)}\left(t\right)-1\right]}^{g}{\left(1+a_{ASL}L\right)}^{\gamma }, \end{array}$$

where $k_{g}^{\left(j\right)}$ is the temperature-dependent rate constant

(17) $$\begin{array}{c} k_{g}^{\left(j\right)}\left(T^{\left(j\right)}\right)=k_{g,eff}\exp\left(-\frac{E_{A,g}}{R_{g}T^{\left(j\right)}}\right), \end{array}$$

where the pre-exponential factor $k_{g,eff}$, is a constant model parameter. On the other hand, the dissolution rate in undersaturated solution is found to be

(18) $$\begin{array}{c} D_{k}^{\left(j\right)}\left(t,L\right)=k_{diss}\left[S-1\right]. \end{array}$$

New particles are born in a supersaturated solution due to primary and secondary nucleation. The secondary nucleation of enantiomer *k* takes place predominantly if crystals of that enantiomer are already present. It can be expressed as

(19) $$\begin{array}{c} B_{sec}^{\left(j\right)}\left(t\right)=k_{b,sec}^{\left(j\right)}\left(T\right){\left[S_{k}^{\left(j\right)}-1\right]}^{b,sec}{\left[\mu_{3,k}^{\left(j\right)}\left(t\right)\right]}^{n_{\mu 3}}, \end{array}$$

(20) $$\begin{array}{c} k_{b,sec}^{\left(j\right)}\left(T\right)=k_{b,sec,eff}\exp\left(-\frac{E_{A,b}}{R_{g}T^{\left(j\right)}}\right), \end{array}$$

where $\mu_{3,k}$ is the third moment of the enantiomer *k*. The temperature-dependent rate constant $k_{b,sec}$ is expressed by an Arrhenius-type equation. The primary nucleation takes place in the clear solution

(21) $$\begin{array}{c} B_{k,prim}^{\left(j\right)}=k_{nuc}{\left[S_{k}^{\left(j\right)}-1\right]}^{b_{nuc}}\Psi \left(t_{ind}\right), \end{array}$$

where induction time $t_{ind}$, defined as the time difference between reaching the initial degree of supersaturation and the point of first measured concentration decrease in the liquid phase under seeded conditions, is introduced that describes the experimental observations reasonably well [11]. Ψ is the sigmoid function where the steepness is determined by the second parameter ϕ

(22) $$\begin{array}{c} \Psi \left(t_{ind}\right)=\frac{1}{2}+\frac{1}{\pi }\arctan\left[\phi \left(t-t_{ind}\right)\right]. \end{array}$$

All the required parameters are listed in Table A1 in the Appendix. The resulting PBEs are solved numerically for the moments of distribution using quadrature method of moments (QMOM) [31]. QMOM transforms the PBEs to a set of ordinary differential equations (ODEs) describing the evolution of moments of distribution. QMOM has been chosen for solving the PBEs as the method of moments (MOM) is not able close the moment chain for this system, a common issue encountered when growth rate is a nonlinear function of crystal size. The resulting set of ODEs coupled with the mass balance equation are solved in Matlab using standard ODE solvers.

## 4. Results and Discussion

### 4.1. DL-threonine/H_2_O as Model System

The well-investigated DL-threonine/H_2_O is chosen as a model system for these studies [11]. The kinetic parameters for DL-threonine are identical for both the enantiomers. For the CPC-MSMPR configuration, two MSMPR vessels of volume V=0.45 L are connected together with exchange of liquid phase. The vessels contain racemic liquid phase at $36{\;}^{\circ }$C ($T_{sat}=45{\;}^{\circ }$C) with homochiral solid phase. The feed streams with the same conditions are fed to each vessel. In order to the keep slurry volume constant, the same amount of slurry as the feed stream is taken out of the vessels as the product stream. The results obtained from CPC-MSMPR are compared with the conventional CPC and CPC-D configurations. The condition for CPC configuration is similar to CPC-MSMPR except that there are no feed and product streams. For CPC-D, the condition for the crystallization vessel (Tank 1) is same as CPC. However, the dissolution vessel (Tank 2) is at saturation temperature $T_{sat}=45{\;}^{\circ }$C and it contains a racemic solid phase. The process conditions are summarized in Table 1.

Eicke et al. [11] have shown the ability of the model to predict the preferential crystallization process within the temperature range considered in these studies. Here, we first investigate the evolution of the solute mass in the liquid and solid phases for all the process configurations. We also investigate and compare the performance of each configuration in terms of productivity and yield. The productivity of the CPC-MSMPR is defined as

(23) $$\begin{array}{c} Pr_{k}^{\left(j\right)}=\left({\dot{m}}_{k,S,prod}^{\left(j\right)}-{\dot{m}}_{k,S,feed}^{\left(j\right)}\right)ee_{S}^{\left(j\right)}, \end{array}$$

(24) $$\begin{array}{c} k=\left\{\begin{array}{cc} E_{1}\left(L-\mathrm{threonine}\right) & \;\mathrm{for}\;j=T1\\ E_{2}\left(D-\mathrm{threonine}\right) & \;\mathrm{for}\;j=T2 \end{array}\right. \end{array}$$

where ${\dot{m}}_{k,S,prod}^{\left(j\right)}$ and ${\dot{m}}_{k,S,feed}^{\left(j\right)}$ are the solid crystal mass flow rates in the product stream and feed stream, respectively, and $ee_{S}^{\left(j\right)}$ is the enantiomeric excess, which is used as a weighting factor in the definition of productivity so that purity of the products is taken into account. $ee_{S}$ is defined as

(25) $$\begin{array}{c} ee_{S}^{\left(j\right)}=\frac{\left|m_{k,S}^{\left(j\right)}-m_{k*,S}^{\left(j\right)}\right|}{m_{k,S}^{\left(j\right)}+m_{k*,S}^{\left(j\right)}}\times 100\%,\\ k*\neq k\;\mathrm{with}\;k,k*\in \{E_{1},E_{2}\}, \end{array}$$

where $m_{k,S}^{\left(j\right)}$ is the mass of the solid phase of enantiomorph *k* in the crystallizer. For the CPC and CPC-D configurations the equivalent productivity for comparison is defined as

(26) $$\begin{array}{c} Pr_{k}^{\left(j\right)}=\left(\frac{m_{k,S}^{\left(j\right)}-m_{k,seed}^{\left(j\right)}}{t}\right)ee_{S}^{\left(j\right)}. \end{array}$$

It is to be noted that for the CPC-D configuration, $m_{k,seed}=0$ for j=T2, as seed is only used in the crystallization tank. In the dissolution tank, a racemic solid is used which is not considered as investment while calculating productivity. The another performance indicator used is the yield of the process which is defined for the CPC-MSMPR configuration as

(27) $$\begin{array}{c} Y_{k}^{\left(j\right)}=\left(\frac{{\dot{m}}_{k,L,feed}^{\left(j\right)}-{\dot{m}}_{k,L}^{\left(j\right)}}{{\dot{m}}_{k,L,feed}^{\left(j\right)}}\right)ee_{S}^{\left(j\right)}, \end{array}$$

where ${\dot{m}}_{k,L,feed}^{\left(j\right)}$ and ${\dot{m}}_{k,L,feed}^{\left(j\right)}$ are mass flow rates of the enantiomorph *k* in the liquid phase in the feed stream and in the product stream, respectively. For the CPC and CPC-D configurations, the yield is defined as follows

(28) $$\begin{array}{c} Y_{k}^{\left(j\right)}=\left(\frac{m_{k,L,feed}^{\left(j\right)}+m_{k,R}-m_{k,L}^{\left(j\right)}}{m_{k,L,feed}^{\left(j\right)}+m_{k,R}}\right)ee_{S}^{\left(j\right)}, \end{array}$$

where $m_{k,R}$ is the mass of the enantiomorph *k* in the racemate. $m_{k,R}$ is nonzero only for Tank 2 in the CPC-D configuration.

### 4.2. Liquid and Solid Phase Mass Evolution

**Figure 2:**
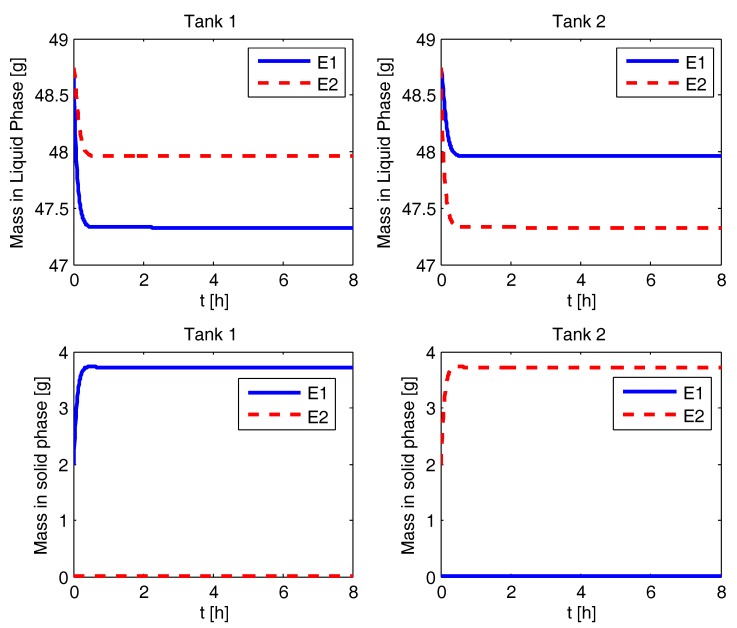
Evolution of mass in liquid and solid phases for the coupled preferential crystallization in MSMPR crystallizers (CPC-MSMPR).

**Figure 3:**
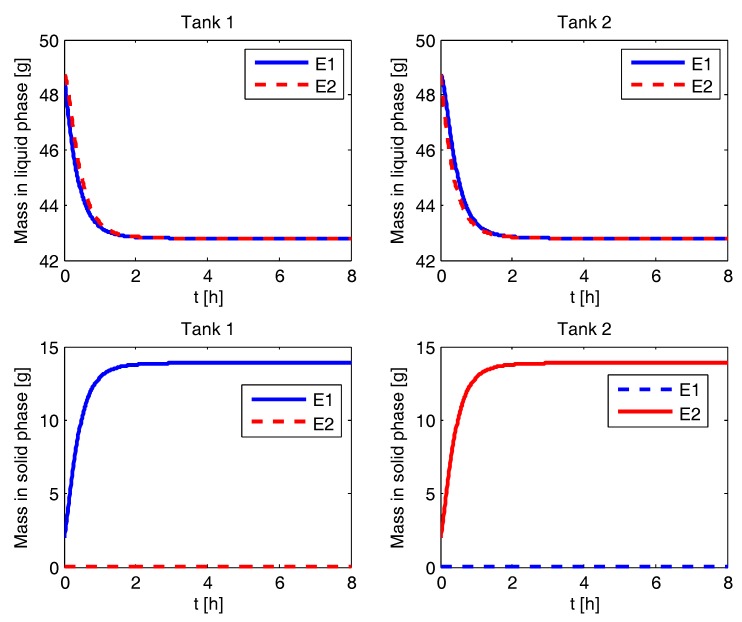
Evolution of mass in liquid and solid phases for the coupled preferential crystallization (CPC) configuration.

**Figure 4:**
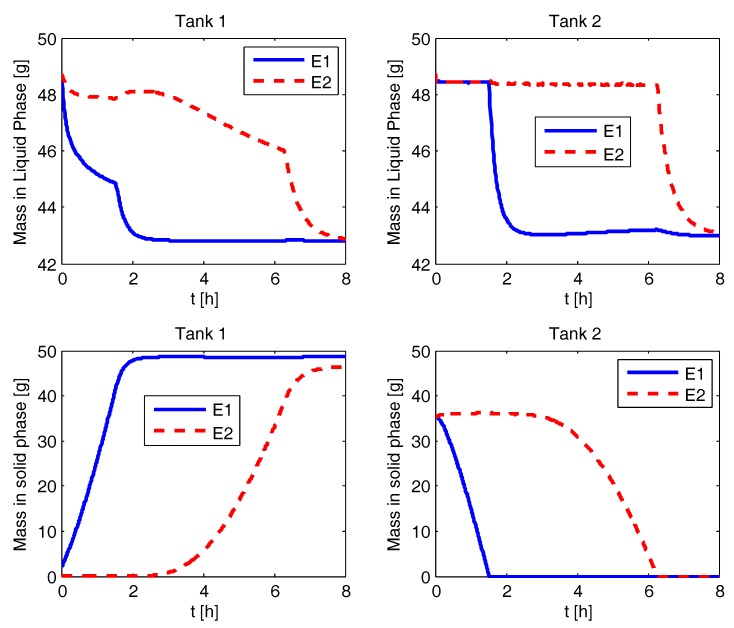
Evolution of mass in liquid and solid phases for the coupled preferential crystallization-dissolution (CPC-D) configuration.

The simulation results for various process configurations are presented in this section. In Figure 2, the results for CPC-MSMPR are presented where the feed and product removal rate used is 80 mL min${}^{-1}$. The solid mass of pure enantiomer increases from seed mass of 2 g to the steady state value of 3.7 g in both the tanks. The nucleation of the counter enantiomer is almost completely suppressed. The racemic liquid phase starts with equal amount of both the eanatiomers. In a time of about 0.5 h, the mass of the enantiomers in the liquid phase reaches a steady state of two different values. The enantiomer which is being enriched in the solid phase has a lower steady sate mass in the liquid phase. The mass evolution profiles for the CPC configuration, shown in Figure 3, is similar to the CPC-MSMPR except the steady sate mass in the liquid phases is the same for both the enantiomers. This is because there are no feed and product streams in this configuration and thus the sufficiently high exchange of liquid phases ensures that the mass of the two enantiomers is the same in the liquid phases. In Figure 4, the mass evolution for CPC-D is presented. The mass of $E_{1}$ in the solid phase of Tank 1 increases with time due to nucleation and growth until it reaches the steady state value at around 2.5 h. As a result, mass in the liquid phase for $E_{1}$ in Tank 1 decreases which is partially compensated due to dissolution of $E_{1}$ in Tank 2. The $E_{1}$ solid in Tank 2 completely dissolves at about 1.5 h, and this results in a sudden increase of depletion rate of $E_{1}$ in the liquid phases at Tank 1 and Tank 2. On the other hand, $E_{2}$ appears in Tank 1 due to primary nucleation after about 2.5 h which results in a decrease of $E_{2}$ concentration in the liquid phase in Tank 1. The rate of depletion of $E_{2}$ in the liquid phases further increases in both the tanks at about 6 h when $E_{2}$ in solid phase in Tank 2 dissolves completely.

### 4.3. Effect of Feed Flow Rate on CPC-MSMPR Productivity and Yield

**Figure 5:**
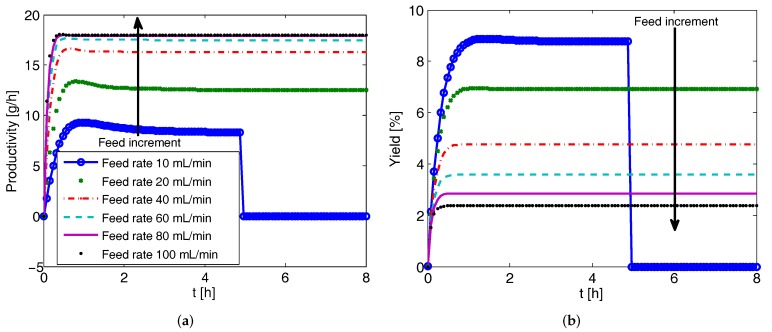
Simulation results showing effect feed flow rate on the productivity and yield for the coupled preferential crystallization in MSMPR crystallizers (CPC-MSMPR) (seed mass 2 g, liquid phase exchange rate 80 mL min${}^{-1}$). (**a**) Increase in feed flow rate up to 80 mL min${}^{-1}$ causes an increase in production to a certain level, beyond which no significant improvement in production is achieved; (**b**) Yield of the process decreases while feed flow rate increases, as increase of feed flow rate reduces the residence time of the solution (for legend please refer to (**a**)).

The effect of feed flow rates on productivity and yield is investigated first for the CPC-MSMPR configuration. The simulation results for Tank 1 are presented in Figure 5. It is to be noted that due to the identical kinetic parameters obtained for both the enantiomers and similar process conditions, the productivity and yield in both the vessels are similar for CPC-MSMPR (and also for CPC) configuration. As can be seen in Figure 5a, the system reaches steady state in about 1.5 h. The increment of feed flow rate up to 80 mL min${}^{-1}$ increases the productivity to a certain level. A further increase in feed flow is accompanied by a negligible increase in productivity. Another important feature in Figure 5a is that the productivity sharply reduces to zero after about 5 h when the feed flow rate is 10 mL min${}^{-1}$. This occurs due to the fact that at low feed flow rates, the residence time of the slurry increases and appearance of the counter enantiomer takes place due to nucleation. This eventually reduces the purity of the crystal product under the specified value of $ee_{S}=99\%$. The optimal feed flow rate is related to the residence time of the seed crystals needed to grow by consuming supersaturation in the MSMPR. Conversely, the yield of the process decreases as the feed flow rate increases as shown in Figure 5b. This is because the increase of feed flow rate decreases the mean residence time and the solution spends less time crystallizing in the crystallizer. Moreover, if the residence time is too long (e.g., with feed flow rate of 10 mL min${}^{-1}$), the purity of the product crystals can be adversely affected by the nucleation of the counter enantiomer. Like many other processes, a trade-off exists between the productivity and yield for the CPC-MSMPR configuration. The feed flow rate of 80 mL min${}^{-1}$ is used while comparing with other configurations as this flow rate provides high productivity. The low yield can be compensated to some extent by recycling the product solution from the crystallizer to the feed stream after racemization so that overall yield increases [32,33].

### 4.4. Effect of Liquid Phase Exchange Rate on CPC-MSMPR Productivity and Yield

**Figure 6:**
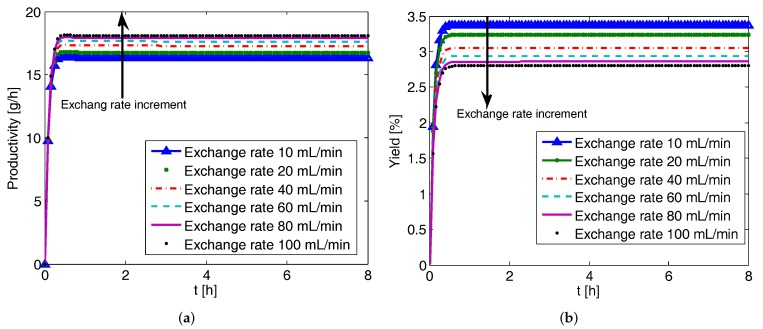
Simulation results showing the effect of the liquid phase exchange rate on the productivity and yield of the coupled preferential crystallization in MSMPR crystallizers (CPC-MSMPR) (feed flow rate 80 mL min${}^{-1}$, seed mass 2 g). (**a**) Increase of liquid phase exchange increases productivity; (**b**) Yield of the processes decreases while the feed flow rate increases, as an increase of the feed flow rate reduces the residence time of the solution.

The liquid phase exchange between the coupled crystallizers helps to compensate the depletion of the preferred enantiomer concentration. The effects of liquid phase exchange rate on productivity and yield are shown in Figure 6. The trends observed are similar to the effect of feed flow rate. As the exchange rate increases, the productivity increases as it enhances the amount of product crystals in the solid phase due to higher rate of crystallization. The return on increased exchange rate diminishes gradually. However, a higher exchange rate would also mean the depletion of the preferred enantiomer in the liquid phase due to crystallization being compensated at a higher rate. The net effect observed is the decrease in yield with the increase of exchange rate.

### 4.5. Effect of Seed Mass on CPC-MSMPR Productivity and Yield

**Figure 7:**
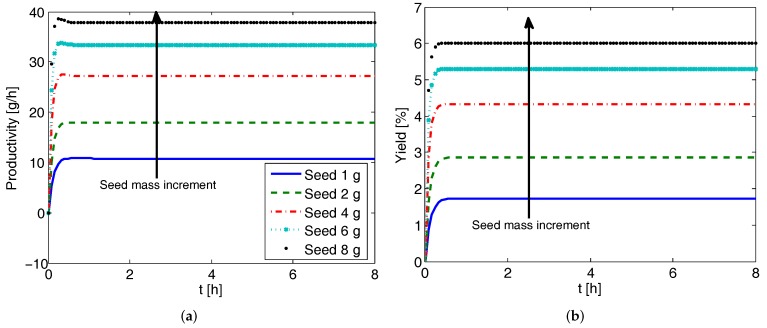
Simulation results showing the effect of seed mass on the productivity and yield of the coupled preferential crystallization in MSMPR crystallizers (CPC-MSMPR) (feed flow rate 80 mL min${}^{-1}$, exchange rate 80 mL min${}^{-1}$). (**a**) Productivity increases with seed mass; (**b**) Yield increases with seed mass (for legend please refer to (**a**)).

Here, we investigate the effect of seed mass on the productivity and yield for the CPC-MSMPR configuration. The simulation results are presented in Figure 7 where the seed mass starting from 1 g is increased to 8 g. As can be seen, both the productivity and yield increase with the increase of seed mass. This can be attributed to the fact that increased seed mass provides increased surface area for the crystal growth. Thus, for the same residence time, more supersaturation will be consumed by the seeds with larger surface area. This has beneficial effect on both the productivity and yield.

### 4.6. Productivity and Yield of CPC and CPC-D Configurations

**Figure 8:**
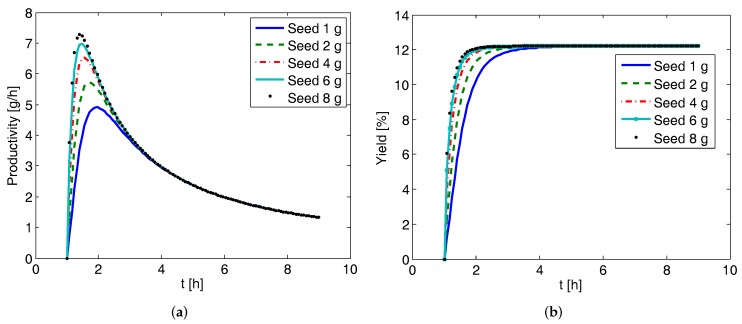
Simulation results showing the effect of seed mass on the productivity and yield of the coupled preferential crystallization (CPC) configuration (exchange rate 80 mL min${}^{-1}$). Final productivity and yield are not affected by seed mass. (**a**) Productivity evolution; (**b**) Yield evolution.

In Figure 8, the effect of seed mass on the productivity and yield for the CPC batch configuration is presented. The liquid exchange rate is kept at 80 mL min${}^{-1}$. Here the downtime (time needed to prepare the vessel for the next batch) is assumed to be $t_{d}$ = 1 h, and thus productivity and yield are zero before 1 h. It can be seen that although after 1 h there are indications of a marginal increment in the productivity and yield, soon the differences become less obvious and after about 3 h there are hardly any differences. This observation is due to the fact that once the supersaturation is consumed at the initial stages of the batch process, the effect of seed mass becomes insignificant as there is not much driving force left. Similar observations were found when the effect of liquid phase exchange rate was investigated (not presented here) for the CPC configuration, i.e., it has insignificant effect on the final productivity and yield.

**Figure 9:**
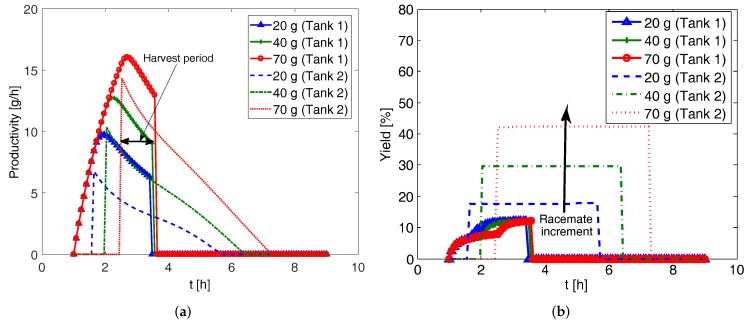
Simulation results showing effect of racemate mass on the productivity and yield of coupled preferential crystallization-dissolution (CPC-D) configuration (seed mass 2 g, exchange rate 80 mL min${}^{-1}$). (**a**) Productivity for Tank 1 and Tank 2; (**b**) Yield for Tank 1 and Tank 2.

Next, in Figure 9 we present the effect of racemate mass on the dissolution tank of the CPC-D configuration. The downtime is taken to be 1 h. The racemate mass is varied from 20 g to 70 g while keeping seed mass at 2 g and liquid phase exchange rate at 80 mL min${}^{-1}$. For each of these considerations, two different curves are obtained in each vessel for both the productivity and yield, since unlike the previous configurations, the process conditions are different in the two vessels in the CPC-D configuration. The sharp changes in the curves are due to the purity requirement of the crystal product which is set to $ee_{S}=99\%$. In Tank 1, the productivity and yield suddenly change to zero because at this time the purity ($ee_{S}$) of the preferred enantiomer goes below 99% due to the nucleation of the counter enantiomer. Conversely, the productivity and yield at Tank 2 suddenly jump from zero to a certain value because the purity of the preferred enantiomer at that tank has just reached 99% due to the dissolution of the counter enantiomer. Thus, in order to ensure the supply of the product crystals from both the vessels, the CPC-D configuration should be harvested during the period enclosed by the time when the product crystal with required purity appears in the dissolution vessel and the time when the purity of the product crystals in the crystallization vessel goes below the required level, as shown in Figure 9a. The productivity and yield increase with the increment of the racemic solid used. However, this increment is accompanied by the decrease in the length of the time period available for product crystal removal. Thus, there is a trade-off between the increase of productivity and the length of harvest period. The effect of seed mass is also investigated (not reported here) and no significant effect is found for the same reason explained for the CPC configuration.

### 4.7. Comparison of Productivities and Yields for Various Configurations

**Figure 10:**
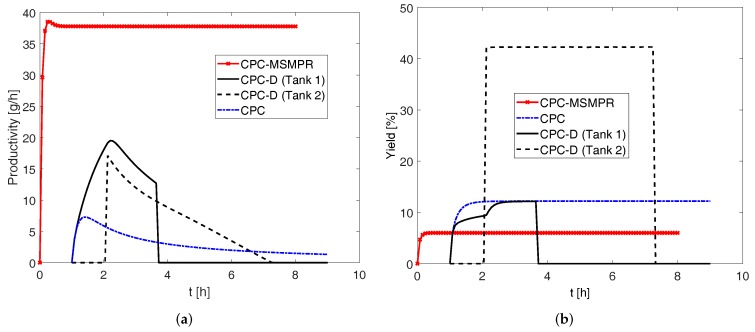
(**a**) Comparison of productivities for various process configurations. Process parameters used are seed mass 8 g, feed flow rate rate 80 mL min${}^{-1}$, exchange rate 80 mL min${}^{-1}$, and racemate mass 70 g. The productivity for coupled preferential crystallization in MSMPR crystallizers (CPC-MSMPR) is found to be the highest. The sudden rise and fall of productivities for the coupled preferential crystallization-dissolution (CPC-D) configuration is due to the use of cut-off purity of $ee_{S}=99\%$; (**b**) Curves showing comparison of yields. The yield of CPC-MSMPR is the lowest. The relatively high yield of CPC-D configuration in Tank 2 is due to the use large amount of racemic solid.

In Figure 10, a comparison of all three configurations is shown in terms of productivity and yield. In this comparison, the process parameters used are seed mass 8 g, feed flow rate 80 mL min${}^{-1}$, and exchange rate 80 mL min${}^{-1}$, for which higher productivities are found in various configurations. The amount of racemic solid used in Tank 2 for CPC-D configuration is 70 g, so that the productivity is high and harvest period is long enough to collect the product crystals with ease.

As can be seen in this figure, the productivity of CPC-D is higher than in the CPC configuration. This result is consistent with Eicke et al. [11]. The sharp decrease of productivity in Tank 1 at about 3.7 h is due to the appearance of the counter enantiomer resulting from nucleation. Conversely, the sudden rise in productivity in Tank 2 at about 3 h indicates the dissolution of the counter enantiomer ($E_{1}$) in Tank 2 so that $ee_{s}=99\%$. In order to ensure that pure enantiomers can be achieved from both the crystallizers of CPCD configuration, the window of time between the dissolution of counter enantiomer in dissolution tank ($ee_{s}=99\%$) and the appearance of the counter enantiomer in the crystallization tank ($ee_{s}=99\%$) should be long enough to allow collection of products. In some process conditions, for example when the seed crystal surface area is not large enough to consume the supersaturation at a given rate, primary nucleation of the counter enantiomer can occur at the crystallization tank before the required dissolution of the counter enantiomer for $ee_{s}=99\%$ (Eicke et al. [11]). Such phenomena lead to the shrinkage of the harvest period to zero and this essentially means that pure enantiomers can be collected from only one tank. In other words, such an operating condition will lead the process to an unattractive region of operation. However, the other two configurations CPC and CPC-MSMPR do not suffer from such limitations. This is due to the fact that both the enantiomers crystallize in separate coupled vessels in CPC and CPC-MSMPR configurations which depletes the supersaturation of the respective enantiomers.

In Figure 10, we can further see that the CPC-MSMPR configuration provides the highest productivity. The increase of productivity found in CPC-MSMPR is almost two-fold compared to the maximum productivity for CPC-D configuration and five-fold compared to the maximum productivity for CPC. Due to the batch configuration, the productivities for CPC and CPC-D configurations are limited by the consumption of the supersaturation which has no provisions to replenish from feed streams. However, this is not the case for the CPC-MSMPR crystallizer where there is a steady supply of racemic solution to the tanks. This also allows the CPC-MSMPR configuration to maintain the high level of productivity and purity for about 8 h, indicating that the process has reached a steady state. Thus, the superiority of the continuous configuration in terms of productivity as compared to the batch configurations has been shown by these simulation results for the system considered. Nonetheless, as can be seen in Figure 10b, this high productivity comes at the cost of low yield. When it comes to yield, CPC and CPC-D configurtions perform better than the CPC-MSMPR configuration. This can be explained by considering the residence time for each configuration. Since the CPC-MSMPR configuration has steady feed flow and product removal, the residence time for the slurry is much shorter (5.64 min) as compared to the CPC and CPC-D configurations (8 h). As a result, the time available for the seed crystals to grow is much shorter for CPC-MSMPR crystallizer and thus a lower yield is obtained. One can choose an appropriate feed flow rate for CPC-MSMPR configuration by balancing productivity and yield based on the priority. However, if there is a provision for recycling the solution obtained from the product stream after filtering the product crystals and subsequent racemization, the low yield of the CPC-MSMPR configuration can be compensated. The overall yield that can be obtained with recycle is equivalent to that of the equilibrium batch process or even greater [32]. However, the increase of yield by using recycle stream can be limited if there is a build-up of impurities within the system [32].

### 4.8. Effect of Nucleation Kinetics on the Performances of Various Configurations

**Figure 11:**
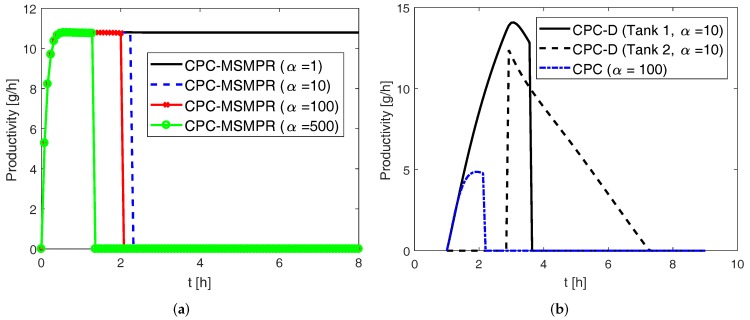
Simulation results showing the effect of nucleation kinetic parameters on productivity in Tank 1 for various configurations. (**a**) Results for the coupled preferential crystallization in MSMPR crystallizers (CPC-MSMPR) with incremental values of α; (**b**) results for coupled preferential crystallization (CPC) and coupled preferential crystallization-dissolution (CPC-D) configurations. The scaling factor α is used to modify the kinetic parameters. With higher nucleation rate (i.e., higher value of α), the purity of the product can go below the cut-off purity of $ee_{S}=99\%$ earlier, thus affecting the productivity.

In the previous sections, experimentally determined kinetic parameters for the DL-threonine-H_2_O system were used in the simulation results. Here we investigate how the nucleation kinetic parameters affect the performance of the various configurations. The secondary and primary nucleation rates for this system are given by Equations (19) and (21), respectively. A scaling factor α is used to scale the parameters $k_{b,sec}^{\left(j\right)}$ and $k_{nuc}$ appearing in those equations. For instance, when α=1, we have the original values of the parameters as shown in Table A1 and when α=10, these two parameters are increased ten-fold. This essentially means that with α=10 the primary and secondary nucleation rates are increased ten-fold in the same process condition. Such a study will be useful in predicting process performance for systems with higher nucleation rates. The simulation results using various values of α for the CPC-MSMPR configuration are shown in Figure 11a where the process parameters used are seed mass 1 g, feed rate 80 mL min${}^{-1}$, and liquid phase exchange 80 mL min${}^{-1}$. As can be seen, the productivities plummet to zero between 1 h and 3 h for α=10,100,500 as it significantly increases the nucleation rate for both the preferred and counter enantiomers, resulting in the product purity dropping below the cut-off purity of $ee_{S}=99\%$. The higher the nucleation rate, the sooner the purity falls below the cut-off value. The simulation results for CPC and CPC-D configurations are shown in Figure 11b where the following process parameters used, seed mass 1 g, liquid phase exchange 80 mL min${}^{-1}$, racemate mass 70 g. As can be seen, similar trends (i.e., drop in productivity) are found with high values of α for the CPC and CPC-D configurations. The harvesting period for the CPC-D configuration has also reduced significantly. However, it is to be noted that a higher value of α=100 is used for the CPC configuration in order to demonstrate the effect of nucleation parameter, as α=10 did not have any noticeable effect . This is because the supersatuation plays an important role in nucleation rate, and in the CPC configuration there are no provisions for the continuous supply of feed slurry or selective dissolution of racemate to compensate for the consumed supersaturation for which both the growth and nucleation rate processes compete. Thus, the effect of the increased value of nucleation kinetic parameters is less prominent for the CPC configuration. For a crystallization system with high nucleation rate, a larger seed mass can be useful in promoting growth and suppressing nucleation.

## 5. Conclusions

Preferential crystallization in vessels coupled via liquid phase exchange is an attractive means for chiral resolution for conglomerate-forming systems. There are various process configurations available for such crystallizations, e.g., batch coupled preferential crystallization (CPC), batch coupled preferential crystallization-dissolution (CPC-D) and coupled preferential crystallization in MSMPR crystallizers (CPC-MSMPR). In this work, first, we investigate the effect of various process parameters on the productivity and yield of the various process configurations with particular focus on the recently proposed CPC-MSMPR. This study is based on process simulation using experimentally verified kinetic parameters from the literature. We then compare the performances of these configurations in terms of productivity and yield. It is found that the productivity of CPC-MSMPR configuration increases as the feed flow rate increases up to about 80 mL min${}^{-1}$. Beyond this flow rate, the increment in productivity is marginal. This phenomena is the result of interplay between system kinetics (nucleation and growth rates) and the residence time of the process. Conversely, the yield of CPC-MSMPR configuration decreases as the feed flow rate increases. Such observations can be attributed to the fact that high flow rate leads to smaller residence time, and the slurry has less time to crystallize. The similar trends are observed when the effect of liquid phase exchange is investigated. It is found that productivity of CPC-MSMPR increases up to an exchange rate of 80 mL min${}^{-1}$, beyond which the return on increased exchanged rate is very limited, while yield decreases with the increase of exchange rate. In the case of the seed mass, it is found that both the productivity and yield increase with the increase of the seed mass as it provides more crystal surface area for the solute molecules in the solution to attach. However, for the CPC and CPC-D configurations, the effect of seed mass is found to be insignificant as the driving force (supersaturation) becomes similar after some time in these batch configurations. A study on the influence of racemic mass in Tank 2 for CPC-D configuration is also performed. As the mass is increased, the productivity increases. However, the time period during which the product crystal has to be collected (after the counter enantiomer dissolves in Tank 2 and before the appearance of counter enantiomer in Tank 1 so that $ee_{S}\geq 99\%$ is maintained in both tanks) narrows down. Thus, one needs to choose the mass of the racemic solid such that productivity can be maximized while the harvest period is long enough for collecting product crystals. Next, the performances of all the process configurations in terms of productivity and yield are compared. The CPC-MSMPR configuration is found to be the most productive among the three configurations, however, with lowest yield. Like many other engineering problems, here is a trade-off relationship between productivity and yield. Nonetheless, based on the priority one can choose appropriate process parameters that serve the required productivity and yield as closely as possible. Moreover, the provision of recycling the solution from product stream to the feed after racemization can be employed to increase the overall yield. Finally, the effect of a growth kinetics parameter is studied to investigate how the systems with high nucleation rates influence the performances of various crystallizer configurations. It is found that the higher nucleation rate of the counter enantiomer can lead to the loss of productivity due to the drop in purity of the product crystals. However, the CPC configuration is found to be less affected as the supersaturation in this configuration is usually lower due to the absence of feed stream.

The insights obtained in these results can be useful in designing a particular process configuration or improving the existing one by manipulating the appropriate process parameters. The presented results will also be helpful in choosing suitable process configuration. For instance, when a continuous supply of pure enantiomers is required, CPC-MSMPR configuration will be preferred. Conversely, if there is no provision for recycle and the separation yield is more important, then the CPC-D configuration will be preferred. Moreover, the analysis presented will be useful in deciding the appropriate feed flow rate, liquid phase exchange rate, and seed mass at the design stage of the process so that the performance of the process can be improved in terms productivity, purity, and yield.

## Figures and Tables

**Table 1:** Process conditions used in simulation studies for coupled preferential crystallization in MSMPR crystallizers (CPC-MSMPR), coupled preferential crystallization (CPC) in batch mode and coupled preferential crystallization-dissolution (CPC-D) in batch mode.

		CPC-MSMPR		CPC		CPC-D	
	Variable	Tank 1	Tank 2	Tank 1	Tank 2	Tank 1	Tank 2
Liquid phase	$m_{\mathrm{rac},L}$	97.48 g	97.48 g	97.48 g	97.48 g	97.48 g	97.48 g
	$m_{H{}_{2}O}$	369.83 g	369.83 g	369.83 g	369.83 g	369.83 g	369.83 g
Solid phase	$m_{E1,S}$ (L-threonine)	2.00 g	−	2.00 g	−	2.00 g	35.00 g
	$m_{E2,S}$ (D-threonine)	−	2.00 g	−	2.00 g	−	35.00 g
Temperatures	$T_{cryst}^{\left(T_{1}\right)};T_{cryst}^{\left(T_{2}\right)}$	$36{\;}^{\circ }C$	$36{\;}^{\circ }C$	$36{\;}^{\circ }C$	$36{\;}^{\circ }C$	$36{\;}^{\circ }C$	$45{\;}^{\circ }C$
Exchange rate	$F_{ex}$	80 mL min${}^{-1}$		80 mL min${}^{-1}$		80 mL min${}^{-1}$	
Feed rate	*q*	80 mL min${}^{-1}$	80 mL min${}^{-1}$	−	−	−	−
Removal rate	*q*	80 mL min${}^{-1}$	80 mL min${}^{-1}$	−	−	−	−

## Appendix A. Model Parameters

**Table A1:** Kinetic and simulation parameters for the ternary system DL-threonine/H_2_O [10,11]. CSD: crystal size distribution.

Parameter	Symbol	Value	Units
constant for density	$K_{1}$	$1.00023\times 10^{-3}$	$m^{3}\;{\mathrm{kg}}^{-1}$
constant for density	$K_{2}$	$4.68\times 10^{-9}$	$m^{3}\;{\left(\mathrm{kg}{\;}^{\circ }C\right)}^{-1}$
constant for density	$K_{3}$	$0.3652\times 10^{-3}$	$m^{3}\;{\mathrm{kg}}^{-1}$
volume shape factor	$k_{v}$	0.1222	-
density of solid threonine	$\rho_{s}$	1250	${\mathrm{kg}\;m}^{-3}$
ideal gas constant	$R_{g}$	8.314	J (K mol)${}^{-1}$
activation energy for growth	$E_{A,g}$	$7.55\times 10^{4}$	J mol${}^{-1}$
activation energy for secondary nucleation	$E_{A,bsec}$	$6.38\times 10^{4}$	J mol${}^{-1}$
dissolution rate constant	$k_{diss}$	$3\times 10^{-5}$	m s${}^{-1}$
growth rate constant	$k_{g,eff}$	$1.24\times 10^{7}$	m s${}^{-1}$
growth exponent	*g*	1.19	-
growth parameter (size-dependent term)	$a_{ASL}$	$2\times 10^{4}$	m${}^{-1}$
growth exponent (size-dependent term)	γ	−0.4	-
secondary nucleation rate constant	$k_{bsec,eff}$	$3.97\times 10^{24}$	m${}^{-3}$ s${}^{-1}$
exponent for third moment	$n_{\mu 3}$	3.0258	-
secondary nucleation exponent	$b_{sec}$	4.8	-
primary nucleation rate constant	$k_{nuc}$	1000	s${}^{-1}$
primary nucleation exponent	$b_{nuc}$	1	-
nucleation induction time	$t_{ind}$	7200	s
slope of sigmoidal function	ϕ	0.01	-
solubility parameter	$m_{sol}$	$1.2\times 10^{-3}$	${}^{\circ }$C
solubility parameter	$b_{sol}$	0.059013	-
solubility parameter	$a_{1sol}$	−0.0780	-
solubility parameter	$a_{2sol}$	−0.1043	-
mean seed size of enantiomer $E_{1}$ (L-threonine)	$L_{mean,E_{1}}$	27,95	μm
standard deviation of seed CSD	$\sigma_{\ln,E_{1}}$	0.3, 0.34	-
mean size of racemate	$L_{mean,rac}$	9	μm
standard deviation of racemate CSD	$\sigma_{\ln,rac}$	0.5	-
